# Assessment of Healthcare System Capabilities and Preparedness in Yemen to Confront the Novel Coronavirus 2019 (COVID-19) Outbreak: A Perspective of Healthcare Workers

**DOI:** 10.3389/fpubh.2020.00419

**Published:** 2020-07-28

**Authors:** Mohammed Zawiah, Fahmi Y. Al-Ashwal, Ramzi Mukred Saeed, Mohammed Kubas, Sara Saeed, Amer Hayat Khan, Syed Azhar Syed Sulaiman, Rami Abduljabbar

**Affiliations:** ^1^Department of Clinical Pharmacy, School of Pharmaceutical Sciences, Universiti Sains Malaysia, Gelugor, Malaysia; ^2^Department of Pharmacy Practice, College of Clinical Pharmacy, University of Al Hodeida, Al Hodeida, Yemen; ^3^Department of Biopharmaceutics and Clinical Pharmacy, School of Pharmacy, The University of Jordan, Amman, Jordan; ^4^Department of Clinical Pharmacy and Pharmacy Practice, University of Science and Technology (UST), Sana'a, Yemen; ^5^Clinical Pharmacy Department, University of Science and Technology Hospital (USTH), Sana'a, Yemen; ^6^Pharmacy Practice Department, Kulliyyah of Pharmacy, International Islamic University Malaysia (IIUM), Kuantan, Malaysia; ^7^School of Dentistry, The University of Jordan, Amman, Jordan; ^8^Advanced Medical and Dental Institute, Universiti Sains Malaysia, Kepala Batas, Malaysia

**Keywords:** Yemen, COVID-19, healthcare facilities, capabilities, preparedness

## Abstract

**Background:** In the past decade, Yemen has witnessed several disasters that resulted in a crumbled healthcare system. With the declaration of COVID-19 a global pandemic, and later the appearance of first confirmed cases in Yemen, there is an urgent need to assess the preparedness of healthcare facilities (HCFs) and their capacities to tackle a looming COVID-19 outbreak. Herein, we present an assessment of the current state of preparedness and capabilities of HCFs in Yemen to prevent and manage the COVID-19 outbreak.

**Methods:** An online survey for HCFs was developed, validated, and distributed. The questionnaire is divided into five main sections: (1) Demographic variables for participants. (2) HCFs capabilities for COVID-19 outbreak. (3) Support received to face the emergence and spread of COVID-19. (4). Current practices of infection prevention and control measures in the HCFs. The last section focused on the recommendations to ensure effective and timely response to this outbreak in Yemen. Descriptive analysis was used to analyze data using statistical package for social sciences (SPSS), version 23.

**Results:** Responses were received from healthcare workers (HCWs) from 18 out of 22 governorates in Yemen. Out of the 296 HCWs who participated in the study, the vast majority (93.9%) believed that the healthcare system in Yemen does not have the resources and capabilities to face and manage a COVID-19 outbreak. Approximately 82.4% of participants rated the general preparedness level of their HCFs as very poor or poor. More specifically, the majority of HCWs rated their HCFs as very poor or poor in term of availability of the following: an adequate number of mechanical ventilators (88.8%), diagnostic devices (88.2%), ICU rooms and beds (81.4%), and isolation rooms (79.7%).

**Conclusions:** The healthcare facilities in Yemen are unprepared and lack the most basic resources and capabilities to cope with or tackle a COVID-19 outbreak. With the current state of a fragile healthcare system, a widespread outbreak of COVID-19 in Yemen could result in devastating consequences. There is an urgent need to provide support to the healthcare workers and HCFs that are on the frontline against COVID-19.

## Introduction

The novel coronavirus (COVID-19) has been declared by the World Health Organization (WHO) as a public health emergency of international concern on January 30, 2020 (1). A few weeks later, on March 11, 2020, WHO declared the COVID-19 outbreak a global pandemic, after the novel coronavirus infected 118,000 individuals in 114 countries (2). As of April 30, 2020, nearly every country in the world has been affected by the virus, and the WHO situation analysis of COVID-19 reported 3,090,445 confirmed cases with 217,769 deaths globally (3). In war-torn Yemen, the first confirmed case of COVID-19 was announced on April 10, 2020, in Hadramout, Yemen's largest province (4). Three weeks later, six confirmed cases of COVID-19 were reported in Yemen, with two deaths (3). The risk of larger outbreaks in Yemen is very high, given the ongoing war and conflicts, political instability and fragmentation, and its fragile health system, where only 45% of the healthcare facilities are fully functioning. The situation in Yemen is further complicated by the presence of high numbers of migrants, refugees and internal displacement of people (IDPs), and concomitant outbreaks of communicable diseases such as cholera, dengue, and diphtheria (4–8). Yemen remains the world's largest humanitarian crisis, with nearly 80 percent of the population requiring some form of humanitarian assistance and protection (4).

The healthcare system in Yemen is largely dependent on the support of international organizations (9). There are 39 health cluster partners [UN agencies, international non-government organizations (NGOs), and national NGOs] that provide support to the primary and secondary healthcare services across the country as of December 2019 (10). However, many gaps in the healthcare system still exist, and the capability and capacity of Yemen's healthcare facilities for facing a widespread COVID-19 outbreak is unknown. Therefore, healthcare facilities (HCFs) preparedness for emergency response and capacities for COVID-19 outbreaks needs to be ascertained. In this study, we describe the current state of emergency response and preparedness for facing COVID-19 in Yemen's healthcare facilities. The study provides a baseline level for preparedness and capacities of the HCFs for facing COVID-19 and allows for future comparative work and intervention progress assessment. Moreover, the results could be utilized by healthcare policy-makers and health cluster partners in designing and providing the appropriate interventions to urgently enhance the preparedness and competency of the HCFs in Yemen, and to ensure their readiness to launch an effective response to prevent, control and manage COVID-19 and future outbreaks.

## Methods

### Study Design and Setting

A cross-sectional study using an online survey-based questionnaire was conducted in Yemen over a period of 2 weeks, opened on March 27, 2020, and closed on April 9, 2020, a day before Yemen's first COVID-19 was revealed. Eligible participants were healthcare workers (HCWs) and administrative personnel working at governmental, private, and non-governmental organizations (NGOs) hospitals. Eighteen out of twenty-two governorates were covered in this survey. Ethical approval was approved by the institutional review board committee at the University of Sciences and Technology, Sana'a, Yemen (ECA/UST189).

### Instrument

An online survey was developed, validated, and distributed to the targeted population. The original draft of the questionnaire was evaluated for face validity by four independent healthcare providers, and modifications were made where appropriate according to the comments and feedback provided. The final version of the questionnaire included five main sections addressing various topics of interest. The first section of this study was demographic data intended to elicit information to describe the respondent. The second part contained: (1) a close-ended question (Yes/No) about whether the healthcare system in Yemen is prepared or not for COVID-19 outbreak; (2) a general Likert-type question (very poor, poor, fair, good, and very good) to rate the capability and preparedness of the healthcare facility they are working in to face COVID-19; (3) 10 specific questions addressing the preparedness level of their healthcare facilities (HCFs). In these 10 questions, HCWs were asked about how their HCFs are prepared in terms of 10 essential competencies for managing the COVID-19 outbreak, including diagnostic devices, mechanical ventilators, intensive care unit rooms and beds, private isolation rooms, personal protective equipment, sufficient trained personnel, adequate knowledge, enough beds in all departments, alternative electricity source, and pre-emptive plans. The third section was about the support received to face the outbreak. Section four assessed the current practices of infection prevention and control measures in the HCFs. The last section addressed the recommendations that should be made to respond to this outbreak in Yemen.

### Survey Implementation and Analysis

Participants were recruited using social media such as Facebook Messengers and WhatsApp; those willing to participate could open a link to initially view the consent form of the study and then proceeding to the survey. Data were collected and aggregated into Microsoft Excel file, exported into statistical package for social science (SPSS) version 21 (SPSS Inc., Chicago, IL, USA), and then analyzed. Descriptive statistics were undertaken using frequency and percentage for qualitative variables and mean and standard deviations for continuous variables. The distribution of various variables was summarized in tables and figures. Chi-square test was used to investigate the differences in preparedness and practice between demographic factors such as hospital types and departments. *P* < 0.05 was considered as statistically significant.

## Results

Responses were received from healthcare workers (HCWs) living in 18 out of 22 governorates of Yemen. The average age of the HCWs (296) that participated in the study was 32.9 years [standard deviation (*SD*): 7.4, range: 20–72], and 240 (81.4%) were male. The majority of participants were general practitioners (25%), specialists (22.6%), and hospital pharmacists (20.3%). Other respondents included nurses (8.8%), individuals with administrative duties (8.1%), laboratory technicians (5.4%), consultants (5.1%), and physician assistants (4.7%). Self-reported years of experience ranged from 1 to 46 years, with the average (SD) being 7.9 (6.5) years. One hundred thirty-three (44.9%) are working in governmental hospitals, 127 (42.9%) in private hospitals, and 36 (12.2%) in NGOs hospitals. Other characteristics are shown in Table 1.

**Table 1:** Healthcare providers' characteristics.

**Category**	**Subcategory**	**F (%)/Mean (SD)**
**Gender**	Male	240 (81.4)
	Female	56 (18.6)
**Age, Years**	Mean ± SD	32.9 ± 7.4
	Minimum	20
	Maximum	72
**Experience, Years**	Mean ± SD	7.9 ± 6.5
	Minimum	1
	Maximum	46
**Specialty**	Consultant	15 (5.1)
	Specialist	67 (22.6)
	GP	74 (25.0)
	Nurse	26 (8.8)
	Hospital pharmacist	60 (20.3)
	Laboratory technician	16 (5.4)
	Physician assistant	14 (4.7)
	Administration	24 (8.1)
**Department**	Emergency	52 (17.6)
	ICU	29 (8.9)
	Pediatric	18 (6.1)
	General/Family medicine	15 (5.1)
	Infectious diseases	10 (3.4)
	Respiratory	7 (2.4)
	Others	165 (55.7)
**Working place**	Governmental hospital	133 (44.9)
	Private hospital	127 (42.9)
	NGO hospital	36 (12.2)
**Governorate**	Sana'a	130 (43.9)
	Aden	38 (12.8)
	Taiz	26 (8.8)
	Ib	21 (7.1)
	Al Hodeida	20 (6.8)
	Others	61 (20.6)

*SD, standard deviation; ICU, intensive care unit; NGOs, non-governmental organization*.

**Figure 1:**
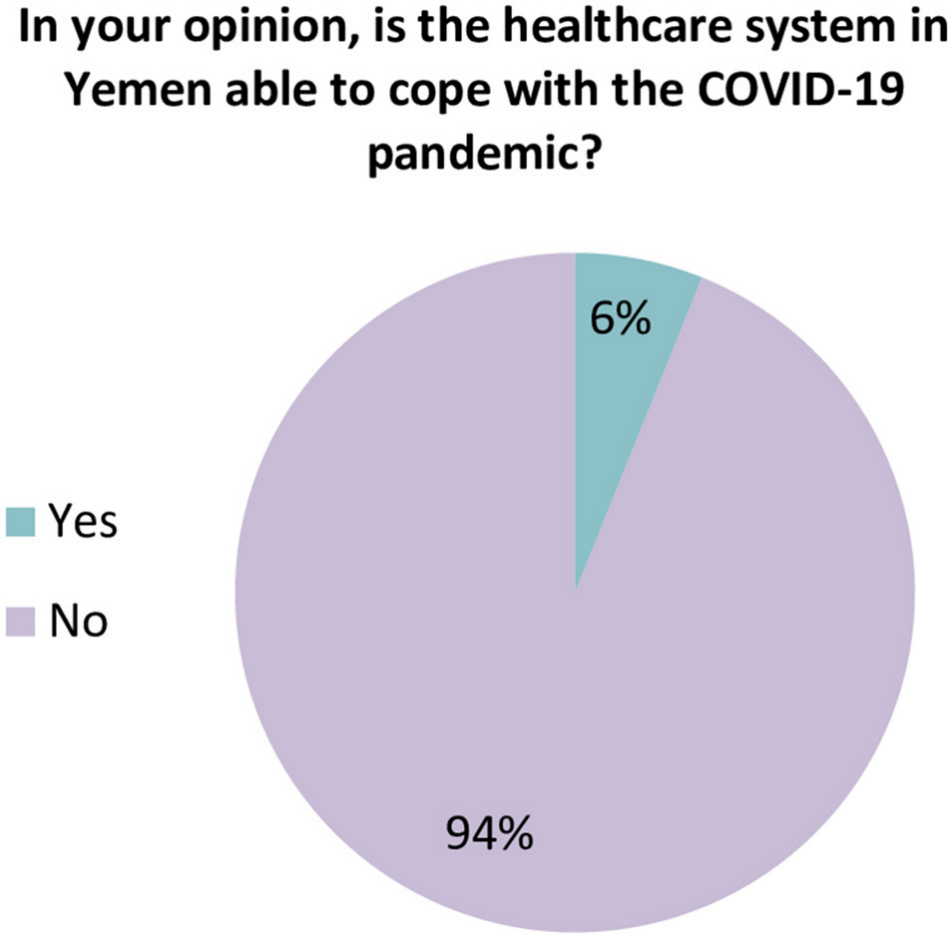
Healthcare workers' perspective regarding the general preparedness of the healthcare system in Yemen.

**Figure 2:**
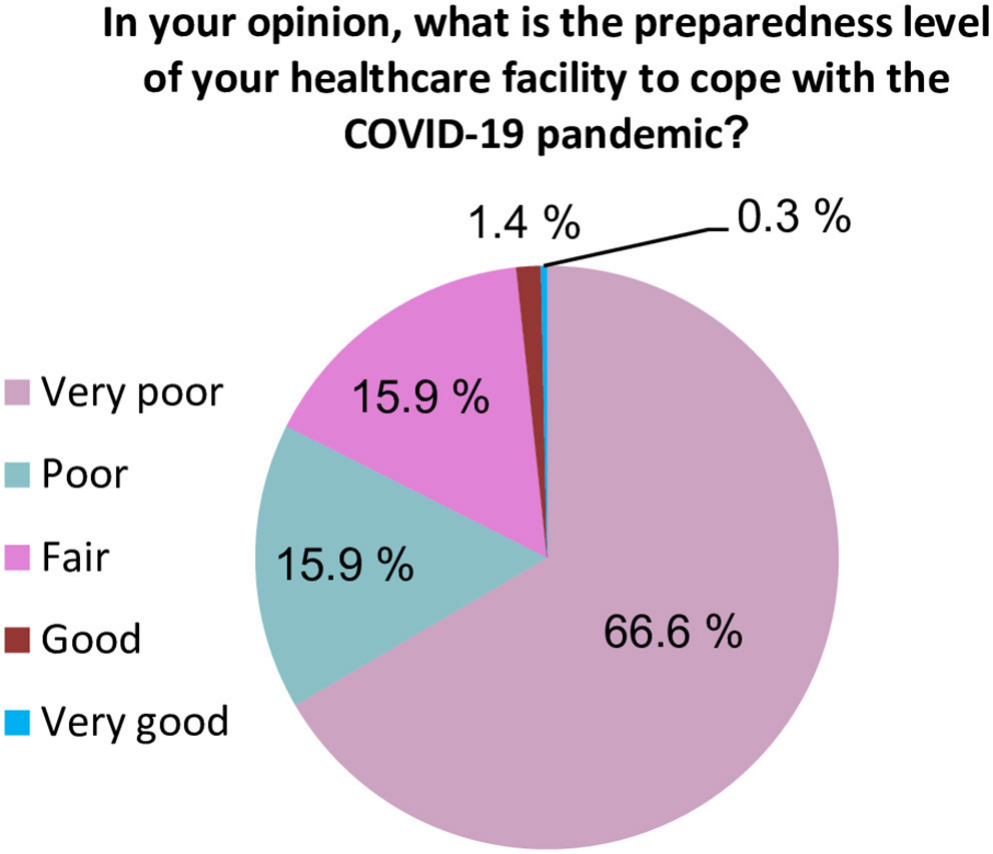
Healthcare workers' perspective regarding the preparedness level of their healthcare facilities.

**Figure 3:**
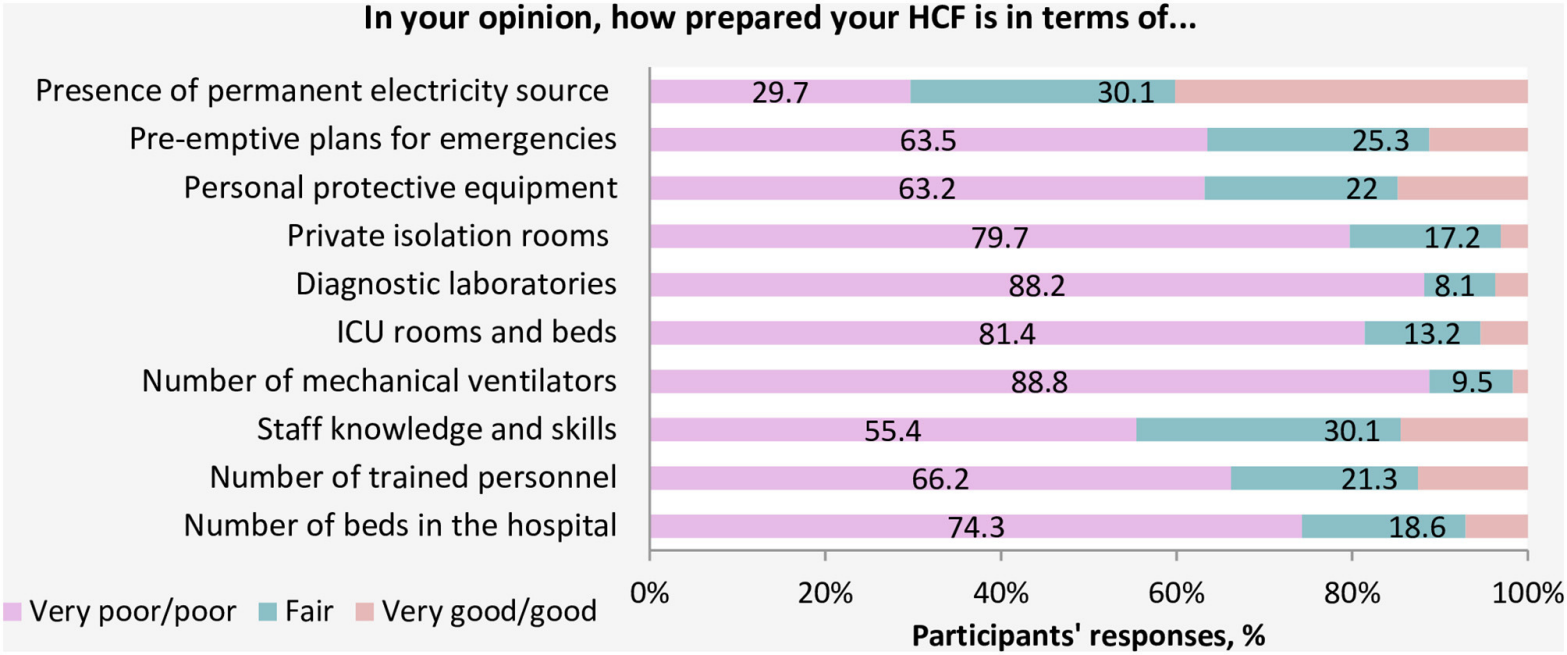
Assessment of essential competencies for HCFs to face COVID-19.

When participants were asked about the readiness of the healthcare system in Yemen, the vast majority of HCWs (93.9%) believed that the current healthcare system in Yemen does not have the resources or capabilities to face and manage the COVID-19 outbreak (Figure 1). Approximately 82.4% of participants rated the general preparedness level of their HCFs as very poor or poor (Figure 2). More specifically, the majority of HCWs rated their HCFs as very poor or poor in term of availability of the following: an adequate number of mechanical ventilators (88.8%), diagnostic devices (88.2%), ICU rooms and beds (81.4%), and isolation rooms (79.7%) (Figure 3). There was a significant difference between hospitals' types in only one preparedness parameter. Governmental hospitals had a much lower level of preparedness in terms of safety equipment in comparison to NGOs and private hospitals, with poor preparedness percentages of 72.9, 52.8, and 55.9%, respectively (*p* = 0.018).

**Figure 4:**
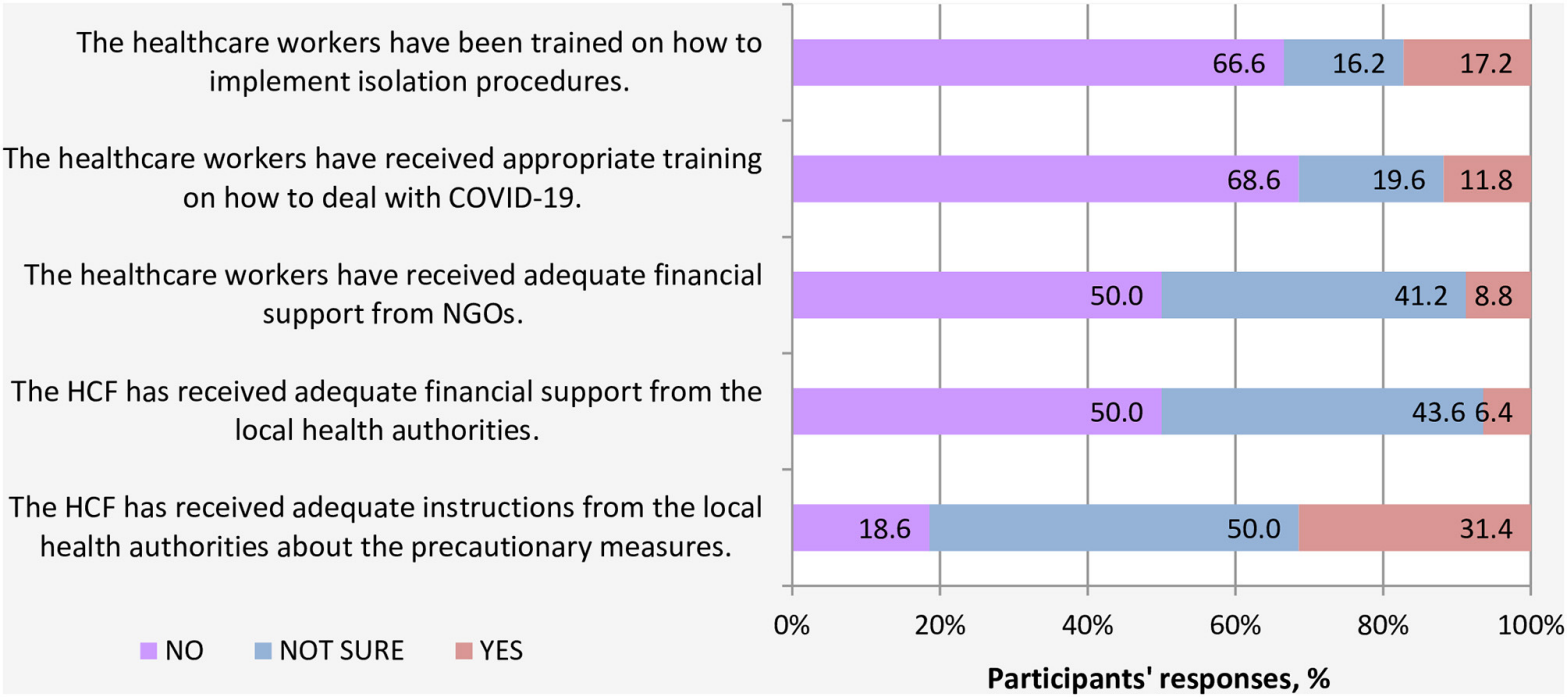
Support received by HCFs to cope with the COVID-19 outbreak.

Regarding the support received from local authorities and NGOs, most HCWs (68.6%) indicated that they did not receive proper training in all aspects related to COVID-19. In this light, a large proportion of participants (66.6%) reported that they had not been trained on isolation procedures. Moreover, half of HCWs indicated that their HCFs did not receive adequate financial support earmarked for addressing and facing COVID-19 pandemic, neither from the local health authorities nor NGOs or international agencies (Figure 4).

**Figure 5:**
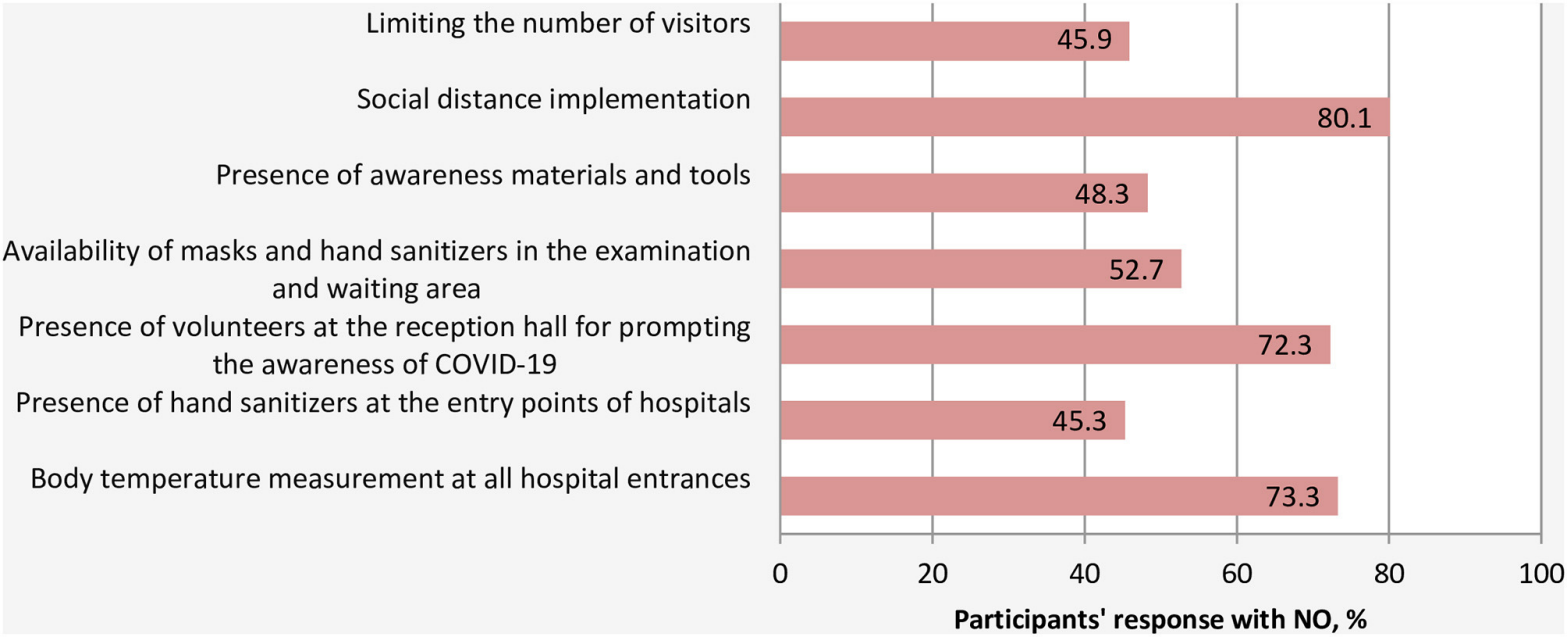
Current practices of preventive and control measures by the HCFs.

With respect to the preventive measures taken by the HCFs to limit and slow the spread of COVID-19, a large proportion of respondents (80.1%) indicated that their HCFs did not implement a social distance strategy, did not measure the temperature of patients and visitor at the entry points of their HCFs (73.3%), and did not have volunteers or employees at the entrance of the hospital to inform and educate the visitors and patients about COVID-19 best practices and preventive measures (72.3%) (Figure 5). There were no significant differences in the practice of preventive measures across hospital departments. However, significant differences were noted between hospitals' types. In this light, the practice of body temperature measurement and the availability of hand sanitizers at all entry points of hospitals were significantly lower in governmental hospitals compared to private and NGOs hospitals with *p*-values of 0.04 and <0.0001, respectively. Similarly, the availability of masks and hand sanitizers in the examination area was much lower in governmental hospitals than other hospitals (*p* < 0.0001).

**Figure 6:**
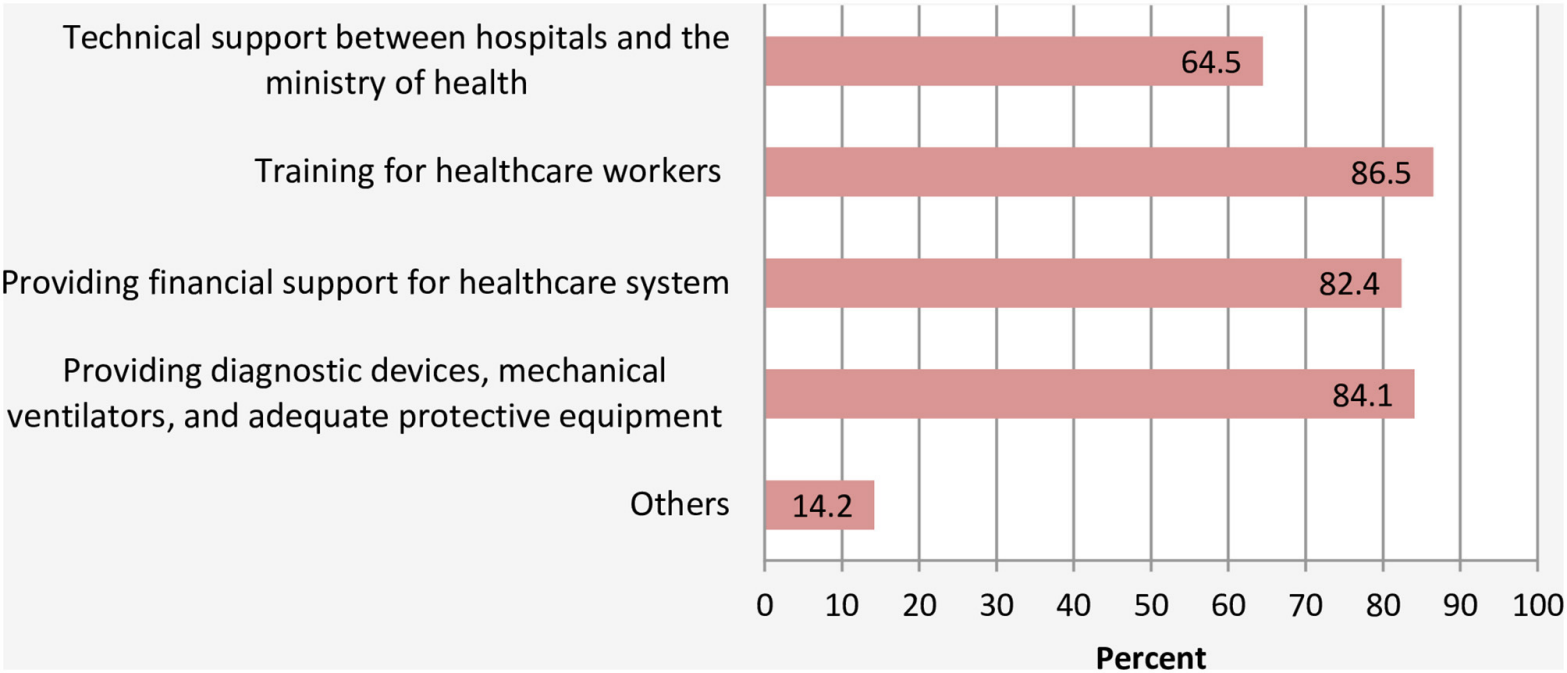
Recommendations that should be made to face COVID-19.

With regards to recommendations, majority of respondents recommended that (1) HCPs should be trained in all aspects of emergency response for COVID-19 outbreak (86.5%); (2) more support is urgently needed for HCFs in the form of diagnostic devices, mechanical ventilators, and adequate protective equipment (84.1%), and (3) financial aid for the HCWs and HCFs to face the outbreak (82.4%) (Figure 6). Some specific recommendations are highlighted below:

- Forming an independent emergency committee of individuals who are not affiliated with any political party to manage the COVID-19 crisis.
- Financial support should be directed to the health care facilities under the supervision of independent organizations.
- Payment of salaries on time and giving financial incentives for all healthcare workers.
- Daily wages workers affected by the infection-control policy should be supported financially.
- Constructing field hospitals to face COVID-19 outbreak.
- Making a management protocol for COVID-19 based on the latest evidence.
- Awareness campaigns for the community using different tools.

## Discussion

Although various measures, by international and national authorities, are ongoing in Yemen to suppress the spread of the outbreak (11), nevertheless, the healthcare system capability and preparedness to combat COVID-19 is still unknown. Several cases of severe acute respiratory syndrome coronavirus 2 (SARS-CoV-2) infection have been reported in the Arabian Peninsula and Middle Eastern region (Saudi Arabia, Egypt, Oman, and Jordan) during the time of data collection of this work with no reported case in Yemen (12). Yemen shares borders with some of these countries, with many people entering the country daily from these borders. These individuals could be potential sources for infection transmission, particularly with the absence of national precautionary steps or strict preventive measures at the borders (13). Given this situation, we aimed to assess the current state of the healthcare system in Yemen.

For demographics, the majority of respondents were male. This can be explained by the fact that males constitute the majority of Yemen's workforce (14). The limited number of consultants participating in this study could be justified by either they were too busy with patients, or they could have limited-time to access social networks. In addition, the number of consultants currently in Yemen has decreased significantly due to migration abroad as a result of the ongoing war and conflict (15). Most of the healthcare workers were from hospitals in Sana'a, the capital of Yemen. This is justified by the fact that Sana'a is the largest city in Yemen and has the highest number of hospitals in the country (16).

The healthcare system capability and general preparedness to face COVID-19 was rated as very poor or poor by the majority of HCWs who participated in the study. This is consistent with international reports, which show that Yemen's healthcare system is fragile and has limited capacity to cope with public health emergencies (17). The country's infrastructure has been destroyed by more than 5 years of conflict. In this light, only <50% of HCFs are fully functioning, leaving a little capacity to respond to COVID-19 or other public health emergencies (11). Since 2015, there have been 142 attacks on hospitals and medical facilities across Yemen (18). By January 2017, four of the Médecins Sans Frontières (MSF) health facilities have been destroyed by airstrikes, resulting in casualties, including deaths, injuries, and ultimately forcing medical staff to leave the country (19).

Very poor availability of essential competencies such as mechanical ventilators, diagnostic devices, ICU rooms, beds, and isolation rooms, and lack of support to HCWs by local authorities is expected. Most of the facilities were left deserted by staff owing to security risks associated with working at those facilities. There is limited medicine, equipment, and personal protective equipment available, and only three testing sites for COVID-19, with a limited number of testing kits, are available in the entire country (Sana'a, Aden, and Al Mukalla). In addition to the war, several other reasons have contributed to pushing the healthcare system in Yemen to the brink of collapsing, including (1) declining public expenditure which due to deterioration in functions of public administration and contraction of country's economy (6); (2) the health system facilities were already overwhelmed by the outbreaks, cholera, and dengue (7, 20).

With regards to infection prevention and control measures, the majority of HCWs felt that their HCFs did not practice the simplest recommended preventive measures to minimize the spread of COVID-19. A significant proportion of HCFs did not adopt a policy for regulating the flow of people to the hospitals by decreasing the number of visitors and limiting the clinic and hospital visits to urgent and emergency cases. This huge gap in practicing these precautionary measures that require a no or low-cost for implementation could reflect the absence of emergency response and infection control plan within the hospitals prior to the outbreak of COVID-19 in Yemen. Social distancing has been identified as a crucial measure for COVID-19 containment and a vital step in slowing the spread of the novel coronavirus, not only in the community (21) but also within the hospitals (22). The risks of visitors with COVID-19 entering HCFs, queuing and staying in overcrowded waiting areas are very real, and the large outbreak of the Middle East respiratory syndrome coronavirus (MERS-CoV) infection in 2015 in South Korea gives a real example and provide us with valuable lessons of how dangerous a single patient exposure can be (23). Therefore, protecting the staff personnel and patients within the HCFs is paramount, and carrying out these proactive measures could play an essential role in the prevention and control of COVID-19.

For recommendations regarding the appropriate interventions to prompt the capabilities of the HCFs in facing COVID-19, the vast majority of respondents agreed that training of healthcare providers, providing them with the appropriate protective equipment, resources, and financial assistance, supporting the health information system for risk communication, and direct support with diagnostic devices and mechanical ventilators are needed. This majority consent could reflect critical shortages of essential medial supply for prevention, control, and management of COVID-19 in Yemen. Other specific recommendations made by the HCWs, include directing the international financial support of COVID-19 containment to an independent committee, which was addressed by many healthcare providers. This reflects a lack of trust toward the local authorities. Others urged the government and international organizations to provide direct financial support to individuals who are being quarantined in the hospitals or being advised for self-isolation by the HCWs. This is very crucial due to the fact that 78% of the population is below the line of poverty, and the majority of them are daily wage workers (24). Thus, implementing such control measures without direct support could exacerbate their financial crisis.

## Limitations

Our study has some limitations. First, the survey did not adequately cover all the hospitals in the country, with low responses were received from some governorates; thus, caution should be exercised in generalizing these findings. Also, due to time constrain and the current emergency state, the questionnaire was only face validated. Moreover, there was no official record-audit, and the data was a perspective of the HCWs. Thus, response bias cannot be rule out as participants may overestimate/underestimate the current capabilities of the HCFs they are working in. Despite these limitations, this is the first study investigating the capabilities and preparedness of Yemen's healthcare system for the COVID-19 pandemic. Also, the study used extensive sources of data, applied a rigorous methodology, and received responses from the main governmental and private healthcare facilities across the country. Finally, our findings are in line with the findings and field reports published by the UN and international organizations (4, 11).

## Conclusion

The healthcare facilities in Yemen are unprepared and poorly equipped to cope with a COVID-19 outbreak. The majority of HCFs do not have enough ICU rooms, beds, isolation rooms, and there are huge deficits of essential medical supplies, testing capabilities, and protective equipment for personnel. Also, proactive measures for prevention and control of COVID-19 are not implemented or adequately enforced. With the current state of a fragile healthcare system, a widespread outbreak of COVID-19 in Yemen could result in devastating consequences. Support and interventions are urgently needed to face COVID-19 pandemic in Yemen.

Based on the current study findings, the following recommendations can be made:

1. Urgent interventions are required to provide PCR devices, diagnostic kits, mechanical ventilators, personnel protective equipment, and other essential medical supply to monitor, manage, and combat a COVID-19 outbreak in Yemen.
2. Training of more healthcare providers on infection control and emergency response is needed to combat the current COVID-19 outbreak.
3. Frontline healthcare personnel should be provided with the appropriate protective equipment to avoid any refusal to work at hospitals designed to receive individuals with COVID-19 infection.
4. Providing salaries on time and financial incentives for all healthcare workers are required to motivate them to engage in treatment and follow-up with COVID-19 patients.
5. Strict emergency plans and preventive measures are needed to be taken by the healthcare facilities.
6. We believe that the different authorities in Yemen should work together with the WHO on this dangerous situation by establishing a national emergency committee for the entire country, and a risk communication system for COVID-19 outbreak must be carried out between the different health authorities throughout the country with the help of international organizations, private, and NGOs-operated hospitals.
7. Building rapid response teams in each city for active surveillance, rapid detection, and management of suspected COVID-19 cases, as this will help in developing and implementing real-time preventive and control measures.
8. The government should be strict and proactive in enforcing the different measures for prevention, control of COVID-19 transmission.

## Data Availability Statement

The original contributions presented in the study are included in the article/supplementary material, further inquiries can be directed to the corresponding author/s.

## Ethics Statement

The studies involving human participants were reviewed and approved by the institutional review board committee at the University of Sciences and Technology, Sana'a, Yemen (ECA/UST189). The patients/participants provided their written informed consent to participate in this study.

## Author Contributions

RS designed the study conception. RS, MZ, FA-A, AK, and SSu analyzed the data and drafted the original manuscript. MK, SSa, RS, RA, MZ, and FA-A participated in data collection and manuscript editing. All authors reviewed and approved the final manuscript.

## Conflict of Interest

The authors declare that the research was conducted in the absence of any commercial or financial relationships that could be construed as a potential conflict of interest.
